# Assessment of heatwave impacts on child feeding practices across 36 low-income and middle-income countries: a cross-sectional analysis

**DOI:** 10.1016/j.lanplh.2025.06.005

**Published:** 2025-07-22

**Authors:** Cheng He, Yixiang Zhu, Jovine Bachwenkizi, Renjie Chen, Haidong Kan, Wafaie W Fawzi

**Affiliations:** **Department of Global Health and Population, Harvard T.H. Chan School of Public Health, Boston, MA, USA** (C He PhD, J Bachwenkizi PhD, Prof W W Fawzi MBBS DrPH); **School of Public Health, Key Lab of Public Health Safety of the Ministry of Education, NHC Key Lab of Health Technology Assessment, IRDR ICoE on Risk Interconnectivity and Governance on Weather/Climate Extremes Impact and Public Health, Fudan University, Shanghai, China** (Y Zhu MS, Prof R Chen PhD, Prof H Kan PhD); **School of Nursing and Public Health, University of KwaZulu-Natal, Durban, South Africa** (J Bachwenkizi); **Department of Environmental and Occupational Health, Muhimbili University of Health and Allied Sciences, Dar es Salaam, Tanzania** (J Bachwenkizi); **Children’s Hospital of Fudan University, National Center for Children’s Health, Shanghai, China** (Prof H Kan); **Department of Epidemiology, Harvard T.H. Chan School of Public Health, Boston, MA, USA** (Prof W W Fawzi); **Department of Nutrition, Harvard T.H. Chan School of Public Health, Boston, MA, USA** (Prof W W Fawzi)

## Abstract

**Background:**

Optimal feeding practices during the first 2 years of life are vital for child survival and growth. Current nutrition programmes in low-income and middle-income countries focus primarily on long-term dietary improvement, overlooking the acute challenges that heatwaves present to daily feeding practices in already nutritionally vulnerable populations.

**Methods:**

By using the Demographic and Health Surveys data, we analysed data from the youngest child aged 6–23 months (including both infants aged 6–11 months and young children aged 12–23 months) in 293137 households across 36 low-income and middle-income countries from 2000 to 2019. Our data came from mothers’ 24-h dietary recall interviews about what foods they fed their youngest child on the previous day and night. Child feeding indicators included minimum dietary diversity (MDD), minimum meal frequency (MMF), and minimum acceptable diet (MAD), following WHO standards. Heatwave exposure was defined using location-specific temperature thresholds (92·5th, 95th, and 97·5th percentiles) with different duration criteria (≥2 or ≥3 consecutive days). Mixed-effects logistic regression models with distributed lag model frameworks were adopted to examine the cumulative effects of heatwaves over 14 days, adjusting for potential confounders.

**Findings:**

Heatwaves significantly disrupted feeding practices among children aged 6–23 months, with the strongest effects observed on the risk of not achieving MDD (odds ratio [OR] for not meeting MDD: 6·19 [95% CI 5·46–7·16] for 3-day heatwaves at 95th percentile threshold). More severe heatwaves additionally compromised the likelihood of achieving adequate meal frequency (OR for not meeting MMF: 2·78 [2·36–3·37] at 97·5th percentile), ultimately affecting children’s ability to receive a minimum acceptable diet (OR for not meeting MAD: 4·66 [3·60–6·62]). These effects persisted up to 2 weeks post-exposure and showed strong negative impacts on consumption of nutrient-rich foods (OR 5·82 [4·44–7·65] for vegetables and vitamin A-rich fruits). Heightened vulnerability to inadequate feeding practices was observed in rural areas, low-income households, families with multiple young children, and those lacking cooling infrastructure (refrigerator or air conditioning), with ORs consistently higher than in their counterpart groups.

**Interpretation:**

Our findings reveal that heatwaves rapidly disrupt child feeding practices, and these disruptions continue for up to 2 weeks. This evidence calls for urgent integration of short-term heat adaptation strategies within existing long-term nutrition programmes, particularly for access to nutrient-rich foods. Improving access to basic cooling facilities, especially in vulnerable communities, is vital for safeguarding child nutrition as global temperatures rise.

## Introduction

Adequate feeding in the first 2 years of life shapes a child’s survival and growth.^1,2^ However, WHO reports that globally, only 44% of infants younger than 6 months receive exclusive breastfeeding, and only one in three children aged 6–23 months achieve minimum dietary diversity requirements for healthy growth.^3^ These challenges are magnified in low-income and middle-income countries (LMICs), where 70% of young children do not meet minimum acceptable diets.^4^ At the same time, these regions are experiencing increasingly frequent and intense heatwaves. The Intergovernmental Panel on Climate Change (IPCC) and other studies project that extreme heat events in LMICs will intensify in both frequency and severity in the coming decades.^5–7^ Heatwaves disproportionately affect vulnerable populations in LMICs due to limited adaptive capacity.^8,9^ Although research has extensively documented heat’s direct health effects and potential to disrupt food systems, we know surprisingly little about how these temperature extremes affect day-to-day child feeding practices—a cornerstone of child health.^10^

Heatwaves could affect infant feeding through multiple pathways, with both immediate and lasting consequences.^11^ Direct physiological effects of heat exposure can reduce infant appetite^12^ and alter feeding behaviours,^13^ while extreme temperatures can disrupt food storage, preparation, and handling practices at the household level.^14^ Heat stress can also impact caregivers’ capacity to maintain regular feeding routines, particularly in households lacking cooling infrastructure.^15^ In addition, these impacts might be particularly pronounced for certain essential food groups: nutrient-rich foods such as fruits and vegetables are more susceptible to heat-related spoilage and often require more complex preparation,^16,17^ yet these foods are crucial for meeting infant nutritional needs.

Current strategies for adaptation to extreme weather in LMICs focus mainly on acute health emergencies among adults, leaving young children’s feeding needs largely unaddressed.^8^ This oversight is particularly concerning as the first 1000 days of life form an important window where poor nutrition can permanently alter development.^18^ Moreover, while existing nutrition programmes in LMICs are mainly established for long-term dietary improvement,^19^ they rarely consider specific strategies for maintaining adequate feeding for young children during shorter-term crisis periods. This gap is most striking in areas where families struggle with basic food storage and unreliable power supply, making it difficult to maintain both food quantity and quality during heatwaves. Moreover, the challenge might particularly be acute for essential foods that provide critical nutrients for infant growth.

This study, therefore, aims to provide evidence on heatwave impacts on children’s feeding practices in LMICs. By analysing nearly 300000 children aged 6–23 months (the youngest child per household, living with their mother who provided feeding care) across 36 LMICs from 2000 to 2019, we assessed the effects of heatwaves on different aspects of infant and young children feeding practices (the variety of foods they eat, how often they are fed, and whether they receive overall adequate diets), examined differential impacts across food groups, and identified key sensitivity and adaptive capacity factors.

## Methods

### Data source

Our study used data from the Demographic and Health Surveys (DHS) that were georeferenced and available for child feeding frequency and quality in the past day and night. The DHS was routinely conducted (3-year to 5-year intervals) in more than 90 LMICs worldwide, collecting information on maternal and child health and sociodemographic characteristics at the household level.^20^ Additional information about case selection from this database is provided in the appendix (p 3).

Data on child feeding practices were derived from the DHS module of child nutrition questionnaires. Based on the indicator definitions,^21–23^ we applied several case inclusion criteria: only the youngest child per household was included, and the child must be aged 6–23 months and living with the mother who provided feeding care. The focus on 6–23 months reflects a critical window for physical and cognitive development. Specifically, this age range includes both infants (6–11 months) and young children or toddlers (12–23 months), who have different nutritional needs and feeding patterns as they progress through key developmental milestones. Adequate nutrition during these transitions can significantly impact long-term health outcomes.^24^ The DHS questionnaire specifically targets this age range because it aligns with WHO’s standardised indicators for infant and young child feeding practices.^23^ This period represents the crucial transition when children move from exclusive breastfeeding (recommended for the first 6 months) to requiring complementary foods alongside continued breastfeeding. During this vulnerable transition, children face heightened risks of malnutrition, stunting, and micronutrient deficiencies if feeding practices are inadequate.^25^ More importantly, by following WHO’s established measurement standards for this specific age group, the DHS ensures collection of internationally comparable data on multiple feeding indicators across all participating countries. The selection of the youngest child provides a reliable representation of current maternal feeding practices within households. The feeding indicators were assessed as binary variables based on dietary intake in the past day and night, indicating whether the diet records met the criteria for minimum dietary diversity (MDD), minimum meal frequency (MMF), and minimum acceptable diet (MAD). MDD is defined as consuming at least five out of eight food groups during the previous day or night, according to WHO standards:^23^ (1) breastmilk; (2) grains, white or pale starchy roots, tubers, and plantains; (3) legumes and nuts; (4) dairy products; (5) flesh foods; (6) eggs; (7) vitamin A-rich fruits and vegetables; and (8) other fruits and vegetables. MMF requirements vary by age:^23^ breastfeeding children aged 6–8 months should receive two or more feeds and those aged 9–23 months should receive three or more feeds, while non-breastfeeding children aged 6–23 months should receive four or more feeds with at least one being solid, semi-solid, or soft food. MAD combines both dietary diversity and meal frequency requirements with specific criteria for breastfed and non-breastfed children.^23^ For breastfed children, both MDD and MMF criteria must be met. For non-breastfed children, MDD, excluding dairy products (four out of six groups), MMF, and at least two milk feeds are required. These indicators were calculated based on the above definitions and detailed feeding records by the Integrated Public Use Microdata Series (IPUMS). The DHS data used in this study are publicly available upon application.

Additionally, from IPUMS, we collected geographical coordinates of living clusters and detailed information on specific food group consumption on the previous day and night of each child. A living cluster is the smallest geographical survey unit, typically representing a census enumeration area containing a group of households. These clusters serve as the best available proxy for a child’s residential location, offering sufficient geographical precision for matching with climate data while maintaining respondent confidentiality through slight coordinate displacement. This approach balances the need for spatial accuracy in exposure assessment with ethical requirements for protecting participant identity in publicly available datasets.^26^ Based on data availability, we further categorised foods into eight groups that closely mirror WHO’s definition of MDD food groups: (1) breastfeeding, (2) dairy products, (3) eggs, (4) meat or organ meat, (5) fish, (6) grains, white or pale starchy roots, and tubers, (7) legumes (beans, peas, nuts), and (8) orange, yellow, or green vegetables, or vitamin A-rich fruits or vegetables. We also collected child characteristics (age, height, weight, and breastfeeding status) and maternal socioeconomic information, including age, living arrangements (living alone or with a partner), and education level. Household characteristics included the number of children younger than 5 years, urban or rural residence, housing status (usual living place or temporary housing), wealth level, and availability of key heat adaptation infrastructure, including usage of refrigerator and air conditioning.

We included all eligible children from 36 LMICs across the Middle East, south Asia, and sub-Saharan Africa from 2000 to 2019. Although the DHS programme has conducted surveys in more than 90 countries, our analysis was limited to these 36 countries because they met our specific data requirements: georeferenced household cluster locations, precise interview dates, comprehensive child feeding records, and complete data on key covariates essential for heat exposure analysis. Details of the DHS survey for each included country are provided in the appendix (pp 5–6).

The publicly available population data used in this study have been reviewed and approved by the Institutional Review Board of ICF International.^27^ Written consent was obtained from the participants. The study followed the STROBE reporting guideline for cross-sectional studies.

### Definition and matching of historical heatwave events

For the temperature data, daily mean temperature data for each living cluster were obtained from ERA5 reanalysis datasets, which provide global climate data at a spatial resolution of 0·25° × 0·25° longitude and latitude (approximately 30 km^2^) and an hourly scale.^28^ ERA5 has been extensively validated and widely employed in climate health research,^29,30^ particularly in studies examining temperature-related health impacts.^29,31,32^ For this study, based on the geographical coordinates of each living cluster, we extracted daily mean temperature time series for each cluster location. To ensure robust long-term analysis, we compiled temperature data from 2000 to 2020.

Heatwaves were defined following the World Meteorological Organization (WMO) guidelines, using location-specific historical temperature distributions.^33^ This approach accounts for inter-annual temperature variations and captures local acclimatisation patterns, as populations typically adapt to their regional temperature conditions. Specifically, following WMO recommendations in their guideline,^33^ we defined heatwaves as periods exceeding specific temperature thresholds for a minimum number of consecutive days. We primarily used two temperature thresholds (92·5th and 95th percentiles) combined with two duration criteria (≥2 or ≥3 consecutive days). These specific thresholds and duration criteria have already been used in other related studies.^34–36^ To comprehensively examine the effects of different heat intensities, we included an additional temperature threshold (97·5th percentile), resulting in six heatwave definitions. This multi-definition framework enables a more robust examination of heat exposure effects across different intensity levels.

For each location-specific matching, since feeding information was recorded for the previous day and night, we matched heatwave exposure for the day before the interview date for each case. Additionally, to assess potential lag effects of heatwaves, we also matched heatwave exposure for the preceding 14 days (lag 0–14). As a sensitivity analysis, we extended this matching to include exposure data for the preceding 20 days to evaluate the robustness of our findings over a longer time period.

### Statistical analysis

For analysing the impact of heatwave events on child feeding practices, we used a mixed-effects logistic regression model.^37^ In this main model, heatwave events were analysed using distributed lag models to account for delayed effects.^38^ A maximum lag of 14 days was selected based on previous studies indicating that while effects emerge immediately, significant impacts typically persist for 2 weeks. The lag-response function was built using a natural cubic B-spline with 4 degrees of freedom and two internal knots placed at equally spaced values in the log scale.^35,36^ Control variables included child characteristics (sex, age, and length-for-age Z score, which are calculated from the child’s height, age, and sex), maternal factors (age, education level), residence type (urban and rural), and household wealth. The selection of these variables is supported by extensive research demonstrating their influence on child feeding practices and nutritional outcomes.^39–41^ We also included fixed effects for the individual years and survey months to control for seasonality and long-term trends. Then, the model was also adjusted for annual mean temperature at the individual cluster for the past 12 months. In addition, a random intercept for each case’s country or region was included to account for unobserved environmental and socioeconomic differences across different geographical settings. This modelling framework was applied consistently across all analyses, including different heatwave definitions for the three feeding indicators and various food groups. For the analysis of breastfeeding, we restricted the sample to currently breastfed infants to avoid potential confounding from voluntary weaning. The model results represent the odds ratios (ORs) of not meeting minimum feeding standards (MDD, MMF, MAD) or not consuming specific food groups on heatwave days compared with non-heatwave days.

Furthermore, guided by the IPCC framework for climate change impact assessment,^42^ we selected several variables for stratified analysis based on sensitivity factors (maternal age, education level, living arrangements, wealth level, urban or rural residence, housing status, and number of children younger than 5 years) and adaptive capacity factors (availability of refrigerator and air conditioning). To assess whether these factors significantly modified the heatwave effects, we compared the estimated relative risks within each stratified factor using multivariate Wald tests, with p values below 0·05 considered statistically significant.^43,44^

Finally, to validate the robustness of our findings against potential methodological concerns and data limitations, we conducted several sensitivity analyses. First, to account for long-term climate effects, we additionally adjusted for the cumulative precipitation for the past 24 months in our main model. Second, we assessed the sensitivity of our results to specific covariates by sequentially excluding different variables from the main model, including child sex, age, Z score, maternal age, education level, household wealth level, country’s gross domestic product per capita, survey year and month, and annual mean temperature. Third, we tested the sensitivity of model specifications in the distributed lag model of heatwave by varying key parameters, including the maximum lag days and degrees of freedom of lag period. Lastly, to investigate the potential confounding effect of humidity, we collected 2 m dewpoint temperature and surface pressure data from ERA5 to calculate relative humidity, which was then incorporated into our main model for sensitivity testing (appendix p 4).

Detailed descriptions of covariates in the main model, stratified analysis, and sensitivity analysis are provided in the appendix (pp 3–4).

### Role of the funding source

There was no funding source for this study.

## Results

**Figure 1:**
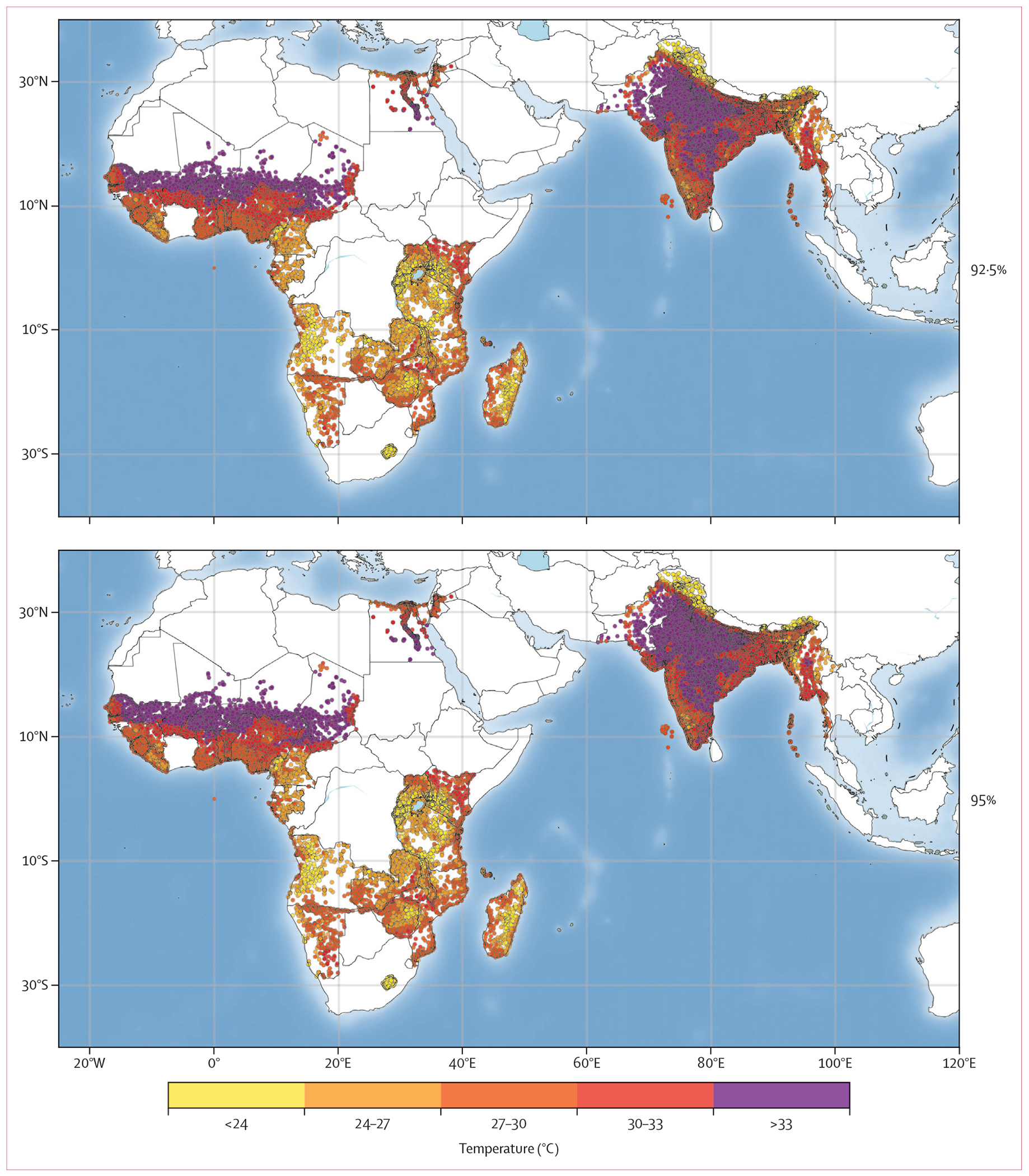

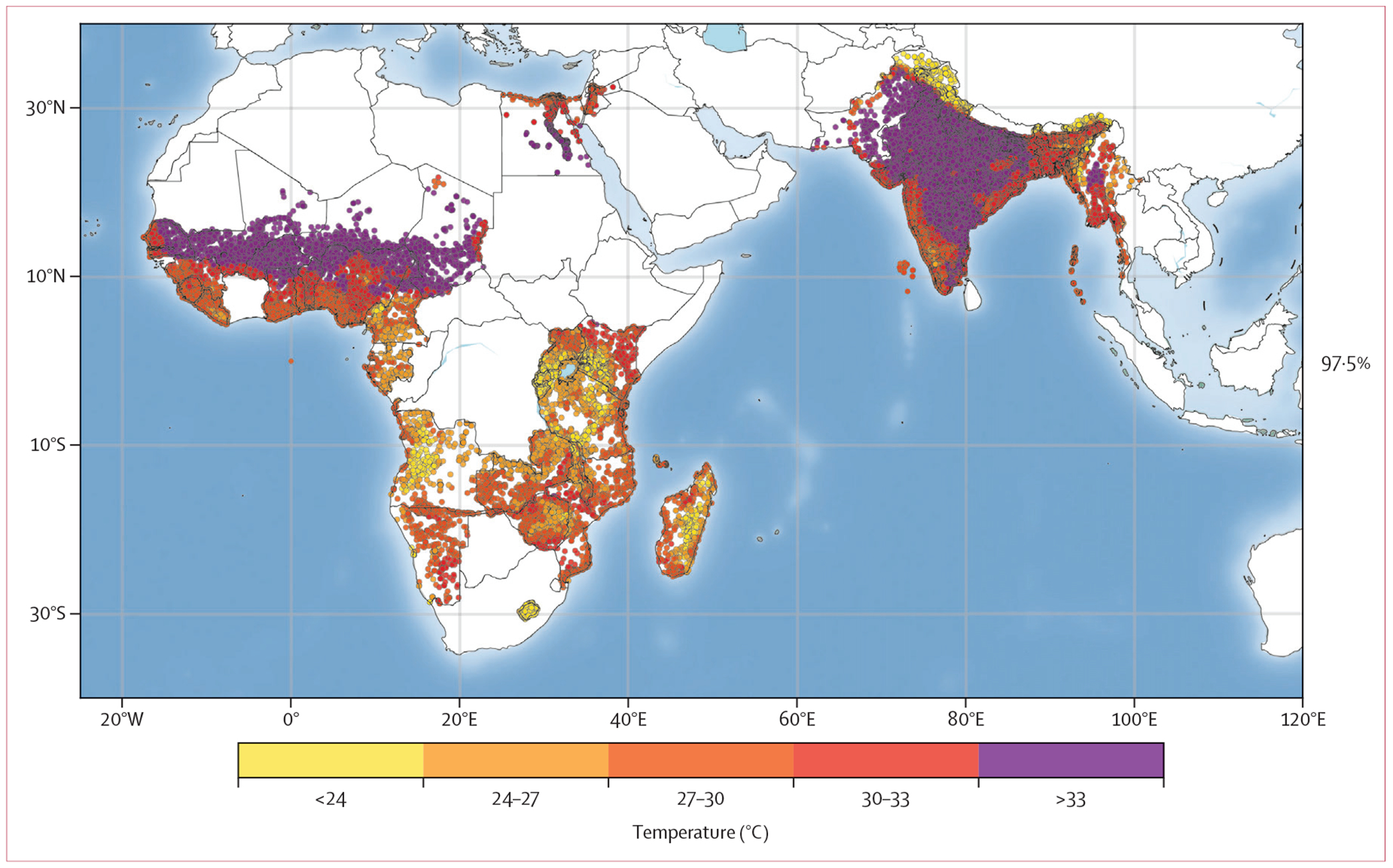
Geographical distribution of study household clusters and location-specific heatwave thresholds (92·5th, 95th, and 97·5th percentiles of daily mean temperature, °C) across the Middle East, south Asia, and sub-Saharan Africa The panel shows the 92·5th, 95th, and 97·5th percentiles of daily mean temperature (°C) during 2000–20 for each household cluster location.

From 2000 to 2019, this study analysed data from infants and young children aged 6–23 months from 293 137 households across 36 LMICs (table, appendix p 7). The median age of children was 14 months (IQR 10–19). Overall, substantial proportions of individuals did not meet the recommended feeding standards: 90·1% did not meet MAD, 79·2% did not meet MDD, and 65·2% did not meet MMF. Sub-Saharan Africa consistently showed higher rates of feeding inadequacy across all three indicators compared with other regions. Notable disparities were observed between urban and rural areas, with rural regions showing significantly higher rates of not meeting feeding standards, particularly for MDD (rural: 80·9% *vs* urban: 74·6%). For specific vulnerability indicators, families with more than three children younger than 5 years showed markedly higher rates of feeding inadequacy, with MAD non-adherence reaching 93·9% (appendix p 7). In addition, households with low wealth levels (below the third quintile) showed higher rates of feeding inadequacy across all indicators (MAD non-adherence: 91·7%), primarily driven by poor dietary diversity (MDD non-adherence: 82·7%). Infrastructure access also played a role, with households lacking refrigeration showing higher rates of MDD non-adherence (81·4%) and consequently higher MAD non-adherence (91·2%). Spearman correlation analysis of individual and household variables showed all correlations were within the range of –0·6 to 0·6, indicating relatively weak correlations between these factors and supporting their independent use in stratified analyses (appendix p 10). Spatially, the heatwave thresholds (defined by the 92·5th, 95th, and 97·5th percentiles of daily temperatures during 2000–20) varied considerably across regions, with notably higher thresholds observed in the Indian subcontinent and regions adjacent to the Sahara Desert (figure 1). Temporally, we observed significant changes in heat patterns over the study period. The median temperature across all study locations increased from 25·1°C (IQR 22·0–26·4) during 2000–05 to 25·4°C (22·8–26·8) during 2015–20. An increase in heatwave frequency accompanied this warming: for the 92·5th percentile threshold (≥2 consecutive days): from a median of 29 (26–33) events per year in 2000–05 to 36 (30–44) events per year in 2015–20; for the 97·5th percentile threshold (≥2 consecutive days): from 5 (3–8) events per year in 2000–05 to 13 (9–17) events per year in 2015–20.

**Figure 2:**
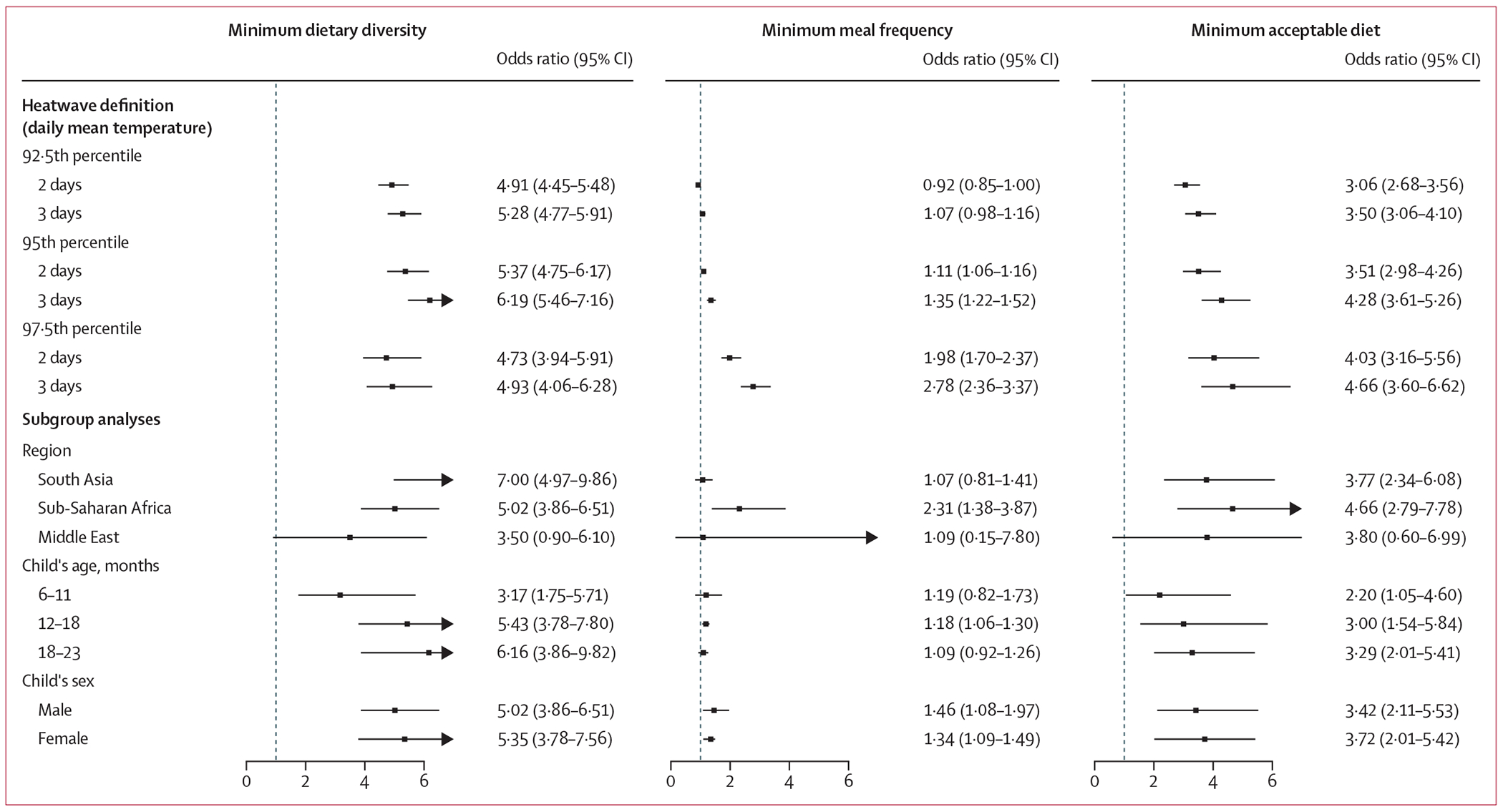
Odds ratios of heatwave effects on the risk of not meeting recommended infant and young child feeding practices across different definitions and population subgroups Point estimates and horizontal lines represent odds ratios and 95% CIs of cumulative effects over 14 days. Effects are shown under different heatwave definitions (92·5th, 95th, and 97·5th percentiles of daily mean temperature on ≥2 consecutive days and ≥3 consecutive days) and according to subgroup analyses by region, age, and sex under the 2-day heatwave definition at the 95th percentile threshold. Odds ratios >1 indicate higher odds of not meeting recommended feeding practices during heatwave days compared with non-heatwave days.

Analysis of the 14-day cumulative effects revealed differential impacts of heatwave exposure on feeding indicators (figure 2). For MDD non-adherence, ORs were consistently high across all heatwave definitions (ORs ranging from 4·73 [95% CI 3·94–5·91] to 6·19 [5·46–7·16]), with the strongest effects observed under 3-day heatwaves at the 95th percentile threshold. The results also showed similar lag patterns across different heatwave definitions, with effects being immediate but decreasing to non-significant levels over 2 weeks post-exposure (appendix p 11).

For MMF non-adherence, significant associations were only observed with higher temperature thresholds and longer durations. At the 97·5th percentile with 3-day duration, the OR reached 2·78 (95% CI 2·36–3·37), while no significant impacts were found under less stringent definitions (92·5th percentile threshold with 2-day duration, OR 0·92 [0·85–1·00] and 3-day duration, OR 1·07 [0·98–1·16]).

The relationship between heatwaves and both MDD and MMF was reflected in the significant associations with MAD non-adherence (OR 4·66 [95% CI 3·60–6·62] for 3-day heatwaves at 97·5th percentile). The lag pattern revealed consistent temporal characteristics across different heatwave definitions, with maximum effects observed approximately 1 week post-exposure and persisting for 2 weeks (appendix p 13).

Sensitivity analyses confirmed that these main findings were robust to adjustments for individual covariates in the main model (appendix p 8), long-term cumulative precipitation, model specifications in the distributed lag model of heatwaves, and short-term relative humidity (appendix p 9).

Subgroup analyses under the 2-day heatwave definition at the 95th percentile threshold (figure 2) showed no significant differences between male and female children in the risk of inadequate feeding practices. While older children aged 18–23 months showed slightly stronger effects on vulnerability to not achieving an acceptable diet, the differences between age groups were not statistically significant. Among the three included regions, comparable levels of feeding disruption were observed on dietary diversity and acceptable diet, except that sub-Saharan Africa showed significantly higher risk (p<0·05) of insufficient meal frequency (OR 2·31 [95% CI 1·38–3·87]) compared with south Asia (OR 1·07 [0·81–1·41]).

**Figure 3:**
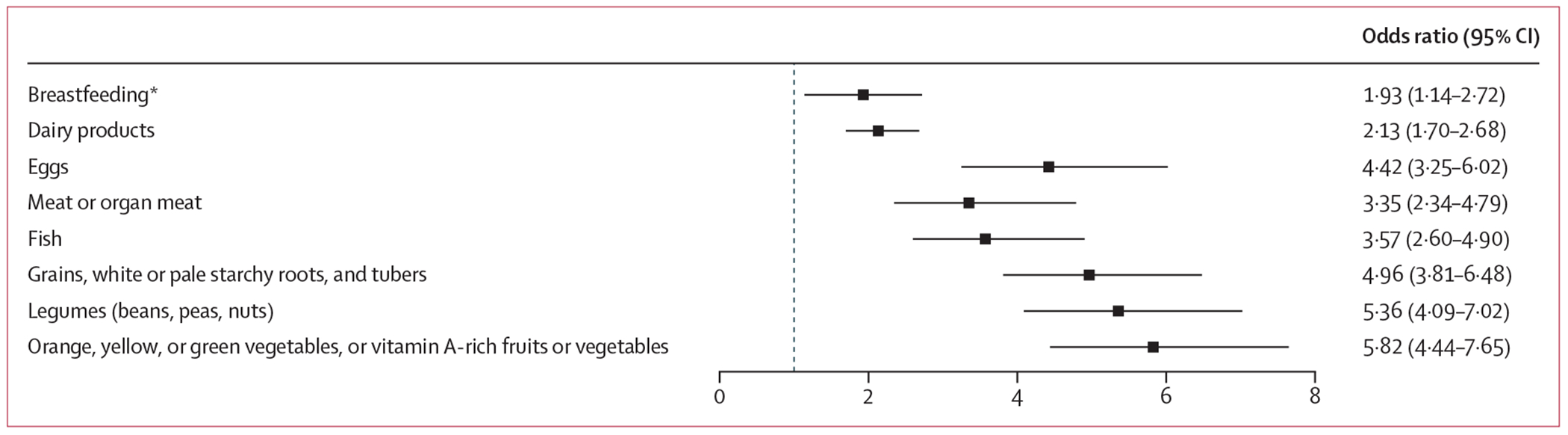
Odds ratios of heatwave effects on the risk of infants and young children not consuming individual food groups Point estimates and horizontal lines represent odds ratios and 95% CIs under 2-day heatwave definition at the 95th percentile threshold. Odds ratios >1 indicate higher odds of not consuming that food group during heatwave days compared with non-heatwave days. *Analysis of breastfeeding was restricted to currently breastfed infants to avoid potential confounding from voluntary weaning.

We examined how heatwaves affected the risk of children not consuming different food groups, comparing feeding patterns on heatwave days versus non-heatwave days. A higher OR indicates that children were more likely to miss consuming that specific food category during heatwave periods compared with normal temperature days. Under the 2-day heatwave definition at the 95th percentile threshold, different food groups showed varying associations (figure 3). The strongest relationships were observed for orange, yellow, or green vegetables or vitamin A-rich fruits or vegetables (OR 5·82 [95% CI 4·44–7·65]), followed by legumes (OR 5·36 [4·09–7·02]) and grains, white or pale starchy roots, and tubers (OR 4·96 [3·81–6·48]). Animal-sourced foods showed relatively lower effects, with eggs showing the highest impact (OR 4·42 [3·25–6·02]), while meat (OR 3·35 [2·34–4·79]), fish (OR 3·57 [2·60–4·90]), and dairy products (OR 2·13 [1·70–2·68]) showed more modest effects. Breastfeeding showed the lowest relative risk among all food types (OR 1·93 [1·14–2·72]).

**Figure 4:**
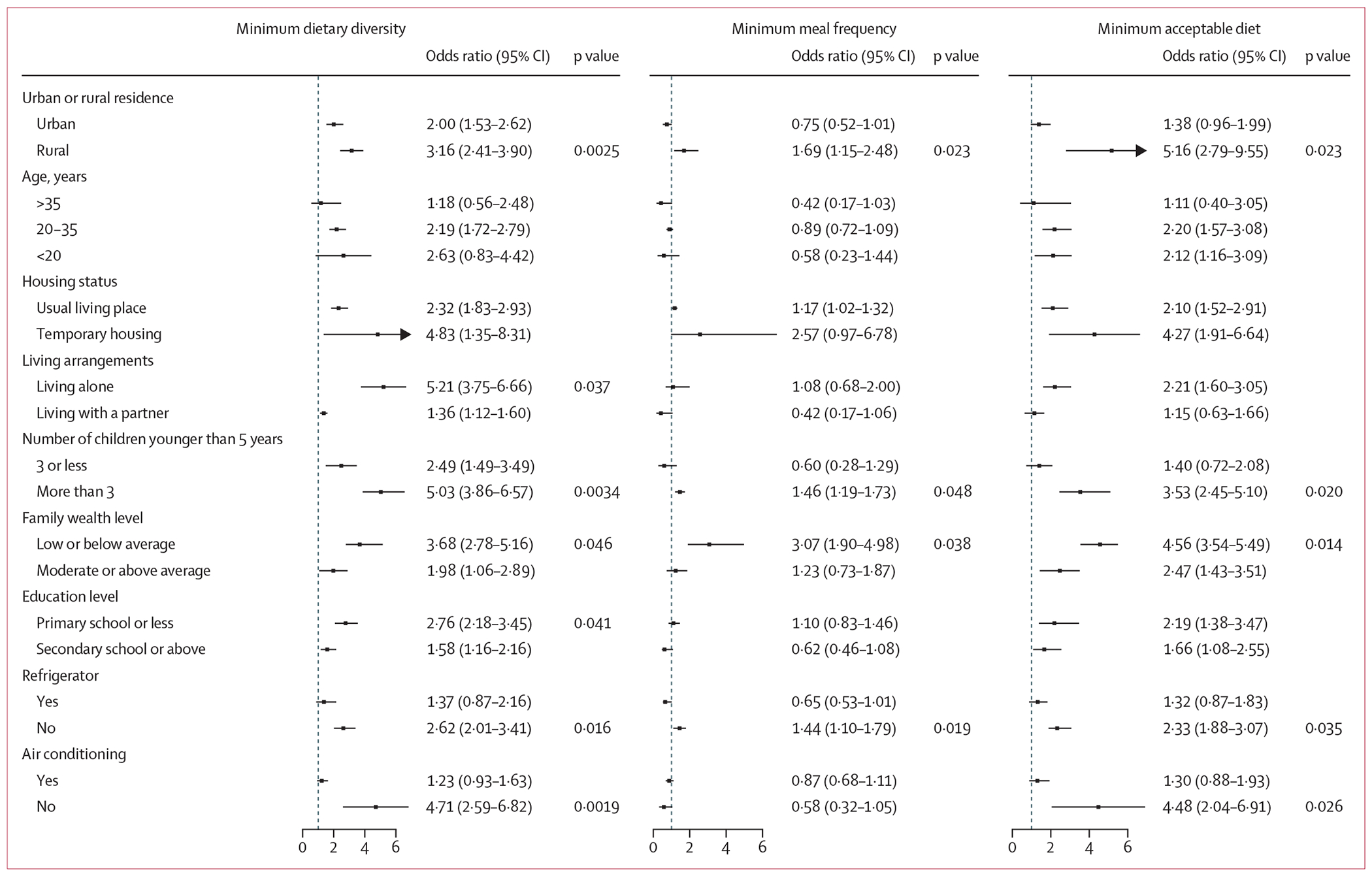
Odds ratios of heatwave effects on the risk of not meeting feeding practice standards among infants and young children by sensitivity and adaptive capacity factors Point estimates and horizontal lines represent odds ratios and 95% CIs under 2-day heatwave definition at the 95th percentile threshold of daily mean temperature. Odds ratios >1 indicate higher odds of not meeting recommended feeding practices during heatwave days compared with non-heatwave days. p values are shown only for factors where subgroup differences were significant (p<0·05, indicating significantly higher odds ratios compared with other categories within the same factor).

Stratified analyses revealed notable variations in heatwave impacts across different sensitivity and adaptive capacity factors (figure 4). Children living in rural areas showed consistently higher vulnerability to inadequate feeding compared with those in urban areas, with significantly larger odds of not meeting standards for MDD (p=0·0025), MMF (p=0·023), and MAD (p=0·023).

Household characteristics significantly modified the heatwave effects. Households with more than three children younger than 5 years showed elevated odds of not meeting all three feeding indicators (dietary diversity: OR 5·03 [3·86–6·57]; meal frequency: OR 1·46 [1·19–1·73]; acceptable diet: OR 3·53 [2·45–5·10]) compared with those with fewer children (p=0·0204). Lower wealth level households exhibited a higher risk of insufficient feeding (MDD: OR 3·68 [2·78–5·16]; MMF: OR 3·07 [1·90–4·98]; MAD: OR 4·56 [3·54–5·49]) compared with wealthier households.

The effects of environmental modifications were particularly evident. Households without air conditioning showed markedly higher risks for not meeting MDD (OR 4·71 [95% CI 2·59–6·82] *vs* 1·23 [0·93–1·63], p=0·0019) and not meeting MAD (OR 4·48 [2·04–6·91] *vs* 1·30 [0·88–1·93], p=0·0264). Similar results were observed for households lacking refrigeration, with significantly higher odds of inadequate feeding across all three indicators (p<0·05).

## Discussion

Our analysis revealed that heatwaves disrupt feeding practices among young children aged 6–23 months. Heat exposure showed a strong association with children not achieving MDD, while severe heat events affected both the consumption of diverse foods and adequate meal frequency, leading to overall inadequate diet quality. These significant disruptions can persist for 2 weeks, with notable impacts on nutrient-rich foods such as vegetables, fruits, and legumes. The impact varied across populations: rural communities, poor households, families with multiple young children, and those without cooling facilities faced greater challenges in maintaining proper feeding practices. This is especially concerning because these same communities already struggle with feeding standards—over 90% of children in our study population across 36 LMICs do not receive adequate diets even under normal conditions.

Our study provides several unique insights that advance current understanding of climate–nutrition relationships. Previous research has primarily focused on long-term climate change impacts on food security and nutritional outcomes,^10,45,46^ and many studies have demonstrated significant associations between various climate exposures and food and nutritional security at both child and household levels.^47–51^ Our findings reveal that even short-term heat exposures can significantly disrupt infant and young child feeding practices—an immediate vulnerability that has been largely overlooked in existing literature.^52^ Moreover, unlike previous studies that mainly examined general nutritional status indicators such as stunting, wasting,^53^ or anaemia,^45^ our analysis of specific feeding practices and food groups provides granular evidence of how heat stress disrupts daily feeding behaviours. The observed persistence of effects for 2 weeks post-exposure further demonstrates that heatwaves can have lasting impacts on child nutrition beyond immediate physiological stress. Importantly, our findings provide a potential mechanistic pathway explaining previously documented poor growth outcomes due to extreme heat exposure, as the disruption in feeding practices we identified could directly contribute to the nutritional deficits observed in other studies.^45,46^ Additionally, our comprehensive assessment of sensitivity and adaptive factors reveals that household-level characteristics and infrastructure play crucial roles in modifying heat vulnerability—aspects that have been underexplored in climate–nutrition research.^53^ These findings suggest that climate adaptation strategies need to consider both immediate heat response and longer-term resilience building in infant and young child feeding practices. The observed disruption of dietary diversity is consistent with previous studies showing that extreme temperatures can affect food availability and dietary quality.^54^ However, our results further demonstrate that such impacts can manifest rapidly during acute heat exposure, rather than only through long-term climate variations as previously documented.

Multiple potential pathways can help explain how heatwaves disrupt child feeding practices. First, physiological mechanisms likely play a substantial role—heat stress may directly reduce infant and young child appetite.^11,52^ This reduction in appetite might occur because the body prioritises thermoregulation over food intake when experiencing heat stress, with metabolic energy being redirected towards cooling mechanisms.^46^ Additionally, hormonal changes during heat exposure, including elevated stress hormone levels and alterations in gut peptides that regulate hunger and satiety,^55^ can further suppress feeding motivation in infants who have limited capacity to verbally express thermal discomfort. Our finding that households with air conditioning experience significantly weaker impacts further supports this physiological pathway. Second, heatwaves can disrupt household food management through multiple pathways: inadequate food storage capacity in the absence of refrigeration leads to more rapid spoilage, particularly affecting perishable nutrient-dense foods such as fruits and vegetables;^56,57^ cooking practices might change during extreme heat to reduce additional thermal stress in homes;^58^ and market access for fresh food might be limited during heat events,^59^ especially for fruits and vegetables that are highly sensitive to temperature fluctuations. In contrast, the impact on animal-source foods might be less pronounced as many of these products can undergo preservation methods such as curing, smoking, or drying that extend shelf life. Research suggests that infants under thermal stress tend to prioritise hydration needs over complex nutritional intake, which could explain our findings of stronger effects on vegetables and legumes compared with more readily consumed animal-source foods.^60^ Third, caregiver capacity can be compromised through both physiological and psychological pathways. Parents experiencing heat stress might have reduced capacity to maintain regular feeding schedules and food preparation routines.^11^ In addition, research shows that women spent considerably less time breastfeeding during the hottest times of the year.^52^ The mechanisms might differ between infants (6–11 months) and young children (12–23 months). Whereas younger infants might experience more direct physiological effects, older children might exhibit different behavioural responses to heat, and caregivers could face distinct challenges in food preparation and feeding routines for different age groups. Overall, these pathways interact with existing vulnerability factors—households with multiple young children face compounded demands during heat stress, while those with limited cooling infrastructure experience more severe indoor thermal stress. This interaction explains our observation that extends beyond traditional socioeconomic vulnerability factors documented in previous studies.^45,53^ Understanding these interconnected pathways is essential for designing effective heat adaptation strategies that address both immediate physiological impacts and broader household resilience factors.

Our findings have specific implications for current heat adaptation strategies and infant nutrition programmes in LMICs. First, while current nutrition programmes in LMICs primarily focus on long-term dietary improvement,^61,62^ our results suggest the need to incorporate heat-specific interventions, such as adjusting the recommended feeding practices during heatwaves and ensuring stable access to nutrient-rich foods during hot seasons. Second, existing early warning systems for heatwaves should extend beyond health emergency responses to include targeted infant feeding support, particularly given the rapid onset and prolonged impacts we observed. Third, our findings on the protective effect of cooling infrastructure indicate that ongoing climate adaptation projects should also consider household-level cooling solutions alongside community cooling centres. Given that traditional adaptation strategies often focus on adult populations, our results emphasise the need to specifically consider infant vulnerability in heat action plans. Moreover, the observed 2-week persistence of impacts suggests that current heat emergency responses, which typically end with the heatwave,^63^ might need to be extended to protect infant nutrition adequately. The differential impacts across food groups also indicate the need for targeted food assistance programmes during heatwaves, particularly focusing on maintaining access to nutrient-rich foods such as vegetables and legumes. Furthermore, given our finding that households with multiple young children show heightened vulnerability, maternal and child health programmes should integrate heat adaptation strategies into their routine services, particularly focusing on practical guidance for maintaining adequate infant feeding during hot seasons. Finally, as climate change continues to intensify, the observed impacts on infant feeding practices could have long-term consequences for human capital development in LMICs, particularly given the critical importance of early-life nutrition for cognitive and physical development. This emphasises the urgency of addressing climate change not only as an environmental challenge but also as a critical determinant of early childhood development and future human capital.

Several limitations of this study should be noted. Although our analysis captured the short-term impacts of heatwaves, the cross-sectional nature of the data limited our ability to examine longer-term adaptations in feeding practices. Additionally, the use of climate reanalysis data might not fully capture household-level heat exposure, particularly indoor temperatures, which could be more relevant for young children’s feeding practices. Another potential limitation is the reliance on maternal reports of feeding practices, which could be influenced by the stress of heat exposure itself; caregivers experiencing thermal discomfort might have altered recall or reporting of feeding behaviours. Although we identified significant effect modifiers, we were unable to fully explore the underlying mechanisms due to data limitations on detailed behavioural responses and physiological measurements (such as short-term food availability changes, cooking behaviour adaptations, or household daily activity adjustments). These limitations point to important directions for future research. Longitudinal studies that track feeding practices across multiple heatwave events could better illuminate adaptation patterns and cumulative impacts. Future studies with more granular data could use quantile regression to examine if heat impacts vary across different levels of dietary intake. Our study uses maternal interviews for feeding data, which excludes children not living with their mothers. However, this limitation is minimal since maternal care is the predominant arrangement for 6–23-month-old children in these countries. Studies incorporating indoor temperature monitoring and detailed behavioural observations could help clarify the relative importance of different impact pathways. Moreover, intervention studies testing specific adaptation strategies, particularly in vulnerable households, would be valuable for developing evidence-based recommendations.

In conclusion, this study provides robust evidence that heatwaves can rapidly disrupt infant and young children’s feeding practices in LMICs, with impacts persisting up to 2 weeks after exposure. The effects are particularly pronounced on disrupting dietary diversity and severe for the provision of nutrient-rich foods, and serious heatwaves additionally compromise meal frequency. The vulnerability to these feeding disruptions is heightened in households with limited adaptive capacity, particularly those lacking cooling infrastructure, suggesting both physiological and behavioural pathways of heat effects. Our findings provide a potential mechanistic pathway explaining previously documented poor growth outcomes due to extreme heat exposure. Given that climate change is expected to increase the frequency and intensity of heatwaves, our findings emphasise the urgent need to integrate short-term heat adaptation strategies into existing long-term feeding support programmes, especially for vulnerable populations. Strengthening household adaptive capacity and extending support beyond immediate heatwave periods will be crucial for protecting infant nutrition in a warming world.

## Supplementary Material

1

## Figures and Tables

**Table:** Distribution of feeding indicators among infants and young children based on included cases in 36 low-income and middle-income countries, 2000–19

	n	Number and proportion not meeting MDD	Number and proportion not meeting MMF	Number and proportion not meeting MAD
Total	293137	232067 (79·2%)	191219 (65·2%)	264151 (90·1%)

Region				
Middle East	16996	11083 (65·2%)	8696 (51·2%)	13894 (81·8%)
South Asia	152613	119936 (78·5%)	102578 (67·2%)	137431 (90·1%)
Sub-Saharan Africa	117363	95696 (81·5%)	74864 (63·8%)	106872 (91·1%)

Child’s age, months				
6–11	98842	85781 (86·8%)	63764 (64·5%)	91235 (92·3%)
12–18	98612	74266 (75·3%)	63319 (64·2%)	87049 (88·3%)
18–23	89866	66350 (73·8%)	58412 (65·0%)	80059 (89·1%)

Child’s sex				
Male	150420	118996 (79·1%)	97822 (65·0%)	135460 (90·1%)
Female	142717	113071 (79·2%)	93397 (65·4%)	128691 (90·2%)

Urban or rural residence				
Urban	81071	60435 (74·6%)	50757 (62·6%)	71532 (88·2%)
Rural	212066	171632 (80·9%)	140462 (66·2%)	192619 (90·8%)

Mother’s age, years				
>35	27626	21650 (78·4%)	17327 (62·7%)	24729 (89·5%)
20–35	245544	193994 (79·0%)	161186 (65·6%)	221359 (90·2%)
<20	19967	16423 (82·3%)	12706 (63·6%)	18063 (90·5%)

All indicators were calculated for infants and young children aged 6–23 months according to WHO standards.

MDD=minimum dietary diversity. MMF=minimum meal frequency. MAD=minimum acceptable diet.

## Data Availability

The demographic and feeding practice data used in this study can be obtained from the DHS through IPUMS and are publicly available at https://www.idhsdata.org/idhs/. ERA5 reanalysis temperature data are publicly available through the Copernicus Climate Change Service (C3S) Climate Data Store at https://cds.climate.copernicus.eu/.

## Research in context

### Evidence before this study

Projections suggest that 80% of children will be born in low-income and middle-income countries (LMICs) in the future. Although research has established that increasing climate variability affects food systems and child nutrition through long-term pathways, the immediate impacts of acute heat exposure on daily feeding practices remain poorly understood. Current nutrition policies in LMICs emphasise long-term dietary improvements but overlook potential short-term disruptions during extreme weather events, creating a critical gap in protecting infant and young child feeding during increasingly frequent heatwaves.

### Added value of this study

Our study provides the first comprehensive assessment of heatwave impacts on infant and young children feeding practices across 36 LMICs. Using standardised feeding indicators and location-specific heatwave definitions, we assessed the impact of short-term extreme heat exposure on feeding adequacy. We identified particularly vulnerable populations and demonstrated the protective role of household cooling infrastructure. Notably, our analysis revealed that heat-related feeding disruptions persist up to 2 weeks post-exposure, with stronger effects on nutrient-rich foods.

### Implications of all the available evidence

Our results suggest that heatwaves can disrupt child feeding through multiple pathways, affecting both the supply of nutrient-rich foods and potentially children’s appetite. These findings suggest that nutrition programmes in LMICs need to incorporate specific heat adaptation strategies, particularly for maintaining access to nutrient-rich foods during and after heat events. The protective effect of cooling infrastructure could be crucial for vulnerable communities beyond immediate heatwave periods.
