# Supplementary material for: Assessment of heatwave impacts on child feeding practices across 36 low-income and middle-income countries: a cross-sectional analysis
Source: Lancet Planet Health. Author manuscript; Available in PMC 2025 Sep 19. (PMC12447361; doi:10.1016/j.lanplh.2025.06.005)
Supplement: 1 [file NIHMS2108326-supplement-1.pdf]

THE LANCET
Planetary Health

Supplementary appendix

This appendix formed part of the original submission and has been peer reviewed. We post it as supplied by the authors.

Supplement to: He C, Zhu Y, Bachwenkizi J, et al. Assessment of heatwave impacts on child feeding practices across 36 low-income and middle-income countries: a cross-sectional analysis. *Lancet Planet Health* 2025. https://doi.org/10.1016/j.lanplh.2025.06.005

Supplementary Materials for

Assessment of heat wave impacts on child feeding practices across 36 low- and middle-income countries: a cross-sectional analysis

Cheng He PhD [1*], Yixiang Zhu MS [2], Jovine Bachwenkizi PhD [1,3,4], Prof. Renjie Chen PhD [2], Prof. Haidong Kan PhD [2,5], Prof. Wafaie W. Fawzi PhD [1,6,7]

1. Department of Global Health and Population, Harvard T.H. Chan School of Public Health, Boston, Massachusetts
2. School of Public Health, Key Lab of Public Health Safety of the Ministry of Education, NHC Key Lab of Health Technology Assessment, IRDR ICoE on Risk. Interconnectivity and Governance on Weather/Climate Extremes Impact and Public Health, Fudan University, Shanghai, China.
3. School of Nursing and Public Health, University of KwaZulu-Natal, Durban, South Africa
4. Department of Environmental and Occupational Health, Muhimbili University of Health and Allied Sciences, Dar es Salaam, Tanzania.
5. Children's Hospital of Fudan University, National Center for Children's Health, Shanghai, China
6. Department of Epidemiology, Harvard T.H. Chan School of Public Health, Boston, Massachusetts
7. Department of Nutrition, Harvard T.H. Chan School of Public Health, Boston, Massachusetts

Supplementary Methods

1. Data sampling methods
2. Covariate
3. Stratified analysis
4. Sensitivity analyses

Supplementary Tables

- Table S1. List of DHS surveys included in the analysis by country, survey year, and sample size
- Table S2. Vulnerability indicators based on included cases in 36 low- and middle-income countries, 2000-2019
- Table S3. Sensitivity analyses of covariate adjustment for heat wave effects on feeding indicators among infants and young children under two-day heat wave definition at the 95th percentile threshold
- Table S4. Sensitivity analyses of model specifications for heat wave effects on feeding indicators among infants and young children under two-day heat wave definition at the 95th percentile threshold

Supplementary Figures

- Figure S1. Spearman correlation matrix of sensitivity and adaptive capacity factors
- Figure S2. Lag patterns of heat wave effects on the risk of not meeting Minimum Dietary Diversity (MDD)
- Figure S3. Lag patterns of heat wave effects on the risk of not meeting Minimum Meal Frequency (MMF)
- Figure S4. Lag patterns of heat wave effects on the risk of not meeting Minimum Acceptable Diet (MAD)

Supplementary References

Methods

1. Data sampling methods

The Demographic and Health Surveys (DHS), receiving support from USAID, systematically gathered comprehensive data from over 90 developing nations at regular intervals spanning 3-5 years[1]. These assessments encompassed various domains including child and maternal wellness, malarial diseases, household abuse, and different health factors. The data collection process implemented a complex two-tiered clustered sampling methodology with stratification[2]. In the first stage, clusters are selected using probability proportional to size sampling within defined strata, where researchers independently select a preset number of clusters with selection probability corresponding to cluster dimensions. In the second stage, field teams conduct a complete enumeration of all dwellings in these selected clusters, after which a specific number of households is identified through systematic sampling with equal probability, ensuring each survey represents a cross-section of the population at that point in time. Qualified interviewers conducted surveys with all women between 15-49 years who had ever been married within these selected households. The sampling framework divided geographic areas into urban and rural classifications across regions, from which clusters were randomly identified, followed by random household selection within each cluster. Statistical analysis confirmed comparable proportional distributions between the sampled population and the source database, validating the representative nature of the collected information and absence of sampling-related bias[3].

2. Covariate

The main analysis model was adjusted for multiple levels of covariates. At the individual level, we included child characteristics (gender, age, and z-score calculated from height, weight, age, and gender[4]) and maternal factors (age categorized as <20, 20-35, and >35 years; education level classified as no education, primary, secondary and higher). At the household level, we controlled for residence type (urban/rural) and wealth quintiles. At the regional level, we included gross domestic product per capita obtained from the World Bank database (https://databank.worldbank.org/indicator/NY.GDP.PCAP.CD/1ff4a498/Popular-Indicators). To account for location-specific factors and better isolate short-term extreme temperature effects, we adjusted for annual mean temperature at each cluster level for the past 12 months, derived from the ERA5 database[5] using cluster-specific geographical coordinates.

3. Stratified analysis

We conducted several stratified analyses to explore how heat wave impacts on infant feeding quality and frequency differed across population subgroups. These included regional setting (urban or rural), maternal characteristics (age: <20, 20-35, or >35 years; resident type: usually live or temporary housing; living arrangements: alone or with partner), household

composition (number of children under 5: ≤3 or >3), maternal education level (primary school or less, secondary school or above), and key adaptation infrastructure (availability of refrigerator and air conditioning). The sample size for each subgroup is presented in Table S2.

4. Sensitivity analyses

We conducted several separate aspects of sensitivity analyses to verify the robustness of our main results. First, we included fixed effects for the annual cumulative precipitation for the past 24 months, derived from the ERA5 database[5] using cluster-specific geographical coordinates. Second, we assessed the sensitivity of our results to specific covariates by sequentially excluding different variables from the main model, including child gender, age, z-score, maternal age, education level, household wealth level, country's gross domestic product per capita, survey year, and month. Third, to test the stability of the distributed lag model in defining heat wave events, we examined several key specifications of the cross-basis function of heat waves: we extended the maximum lag to 20 days to verify whether 14 days was sufficient to capture the full effects, and we varied the degrees of freedom (3-5 df) for the lag structure. Lastly, to investigate the potential confounding effect of humidity on the relationship between heat waves and infant feeding practices, we extracted additional meteorological data from the ERA5 reanalysis dataset. Specifically, we collected 2m dewpoint temperature (°C) and surface pressure (hPa) for each household cluster location, matching these to the day before the interview date to correspond with the 24-hour feeding recall data. Using these parameters, we calculated relative humidity (RH) for the matched date following the August-Roche-Magnus approximation[6,7], which is widely used in meteorological applications. We added this RH variable to our main model to test whether the influence of heat waves on infant feeding practices might be affected by RH.

Table S1 List of DHS survey included in the analysis.

Country	Time of survey	Number (N)	Proportion
Angola	2015	2,240	0.76%
Bangladesh	2007	1,776	0.61%
Bangladesh	2011	2,416	0.82%
Bangladesh	2014	2,378	0.81%
Bangladesh	2018	2,494	0.85%
Benin	2011	3,793	1.29%
Benin	2017	4,070	1.39%
Burkina Faso	2010	2,126	0.73%
Burundi	2010	1,206	0.41%
Burundi	2016	1,997	0.68%
Cameroon	2011	1,874	0.64%
Cameroon	2018	1,459	0.50%
Chad	2014	3,052	1.04%
Comoros	2012	959	0.33%
Egypt	2008	3,580	1.22%
Egypt	2014	5,240	1.79%
Eritrea	2008	846	0.29%
Eritrea	2013	1,614	0.55%
Eritrea	2019	1,491	0.51%
Gabon	2012	1,255	0.43%
Gambia	2019	1,231	0.42%
Ghana	2008	889	0.30%
Ghana	2014	902	0.31%
Guinea	2012	1,043	0.36%
India	2015	75,767	25.85%
India	2019	66,420	22.66%
Jordan	2007	1,616	0.55%
Jordan	2012	2,085	0.71%
Jordan	2017	3,079	1.05%
Kenya	2008	1,787	0.61%
Kenya	2014	6,125	2.09%
Lesotho	2004	515	0.18%
Lesotho	2009	590	0.20%
Lesotho	2014	473	0.16%
Liberia	2007	1,586	0.54%
Liberia	2013	1,165	0.40%
Liberia	2019	874	0.30%
Madagascar	2008	1,721	0.59%
Malawi	2010	1,726	0.59%
Malawi	2016	1,676	0.57%
Mali	2006	3,898	1.33%

Mali	2012	1,465	0.50%
Mali	2018	2,854	0.97%
Mozambique	2011	3,383	1.15%
Myanmar	2015	1,396	0.48%
Namibia	2006	1,453	0.50%
Namibia	2013	696	0.24%
Niger	2012	1,639	0.56%
Nigeria	2008	7,856	2.68%
Nigeria	2013	9,100	3.10%
Nigeria	2018	3,765	1.28%
Pakistan	2017	1,362	0.46%
Rwanda	2010	1,230	0.42%
Rwanda	2014	1,190	0.41%
Senegal	2010	1,388	0.47%
Senegal	2012	2,013	0.69%
Senegal	2014	1,985	0.68%
Senegal	2015	2,059	0.70%
Senegal	2017	3,629	1.24%
South Africa	2016	436	0.15%
Tanzania	2010	2,352	0.80%
Tanzania	2015	3,200	1.09%
Togo	2013	1,099	0.37%
Uganda	2006	855	0.29%
Uganda	2011	749	0.26%
Uganda	2016	1,496	0.51%
Zambia	2007	1,904	0.65%
Zambia	2013	3,884	1.32%
Zambia	2018	2,871	0.98%
Zimbabwe	2005	1,477	0.50%
Zimbabwe	2010	1,674	0.57%
Zimbabwe	2015	1,643	0.56%

Table S2 Vulnerability indicators based on included cases in 36 low- and middle-income countries, 2000-2019

		n	Number and proportion not meeting MDD*	Number and proportion not meeting MMF**	Number and proportion not meeting MAD***
Living arrangements	Living alone	16,278	13,162 (80.86%)	10,643 (65.38%)	14,832 (91.12%)
	Living with a partner	276,857	218,903 (79.07%)	180,574 (65.22%)	249,317 (90.05%)
No. children aged 5 and under	3 or less	196,736	155,738 (79.16%)	124,194 (63.13%)	176,777 (89.85%)
	more than 3	17,758	15,357 (86.48%)	12,504 (70.41%)	16,668 (93.86%)
Wealth level	Low or below (score less than 3)	140,156	115,876 (82.68%)	94,233 (67.23%)	128,578 (91.74%)
	Moderate or above (score higher than 3)	152,981	116,191 (75.95%)	96,986 (63.40%)	135,573 (88.62%)
Education level	Primary school or less	153,224	127,843 (83.44%)	102,532 (66.92%)	140,995 (92.02%)
	Secondary school or above	139,904	104,218 (74.49%)	88,680 (63.39%)	123,147 (88.02%)
Refrigerator	Has	71,586	51,717 (72.24%)	44,046 (61.53%)	62,130 (86.79%)
	No	221,449	180,275 (81.41%)	147,110 (66.43%)	201,935 (91.19%)
Air condition	Has	32,007	25,721 (80.36%)	21,711 (67.83%)	29,234 (91.34%)
	No	173,902	135,568 (77.96%)	115,614 (66.48%)	156,247 (89.85%)
	Unavailable	87,177	70,738 (81.14%)	53,862 (61.78%)	78,624 (90.19%)

Notes: MDD: Minimum Dietary Diversity; MMF: Minimum Meal Frequency; MAD: Minimum Acceptable Diet. All indicators were calculated for infants and young children aged 6-23 months according to WHO standards.

Table S3 Sensitivity analyses of covariate adjustment for heat wave effects on feeding indicators among infants and young children under two-day heat wave definition at the 95th percentile threshold

	Dietary Diversity	Meal Frequency	Acceptable Diet
Main model*	5.37 [4.75, 6.17]	1.11 [1.06, 1.16]	3.51 [2.98, 4.26]
Child gender	5.10 [4.62, 5.69]	1.13 [1.04, 1.23]	3.42 [2.68, 4.61]
Child age	5.20 [4.10, 6.75]	1.13 [1.04, 1.23]	3.41 [2.78, 4.51]
Child z-score	5.60 [4.27, 7.03]	1.10 [1.02, 1.19]	3.62 [2.58, 4.66]
Maternal age	5.36 [4.88, 6.19]	1.11 [1.05, 1.07]	3.48 [2.48, 4.48]
Maternal education level	5.22 [4.03, 6.78]	1.09 [1.02, 1.16]	3.61 [2.79, 4.03]
Household wealth level	5.47 [4.37, 6.59]	1.09 [1.04, 1.14]	3.45 [2.89, 4.11]
Country‘s gross domestic product per capita	5.21 [4.51, 6.01]	1.11 [1.06,1.16]	3.49 [2.87, 4.19]
Survey year	5.23 [4.53, 6.49]	1.10 [1.05, 1.15]	3.52 [2.85, 4.20]
Survey month	5.38 [4.76, 6.20]	1.12 [1.05, 1.20]	3.54 [2.93, 4.15]
Annual mean temperature	5.30 [4.48, 6.51]	1.12 [1.04, 1.20]	3.41 [2.85, 4.31]

Notes: Values shown are odds ratio [95% confidence intervals].

* Main model adjusted for all covariates listed in the table, including child characteristics (gender, age, z-score), maternal factors (age, education level), household wealth level, country's gross domestic product per capita, survey year and month, and annual mean temperature. Each subsequent row represents a model identical to the main model except with the named covariate removed, to assess the sensitivity of results to individual covariates.

Table S4 Sensitivity analyses of model specifications for heat wave effects on feeding indicators among infants and young children under two-day heat wave definition at the 95th percentile threshold

	Dietary Diversity	Meal Frequency	Acceptable Diet
Main model*	5.37 [4.75, 6.17]	1.11 [1.06, 1.16]	3.51 [2.98, 4.26]
Main model + Annual cumulative precipitation	5.37 [4.54, 6.60]	1.17 [1.04, 1.32]	3.49 [2.49, 4.95]
Df for lag-response:3	5.24 [4.34, 6.17]	1.13 [1.02, 1.24]	3.39 [2.53, 4.29]
Df for lag-response:5	5.30 [4.89, 6.10]	1.11 [1.07, 1.15]	3.41 [2.78, 4.51]
Lag period: 20 days	5.61 [3.27, 7.93]	1.15 [1.03, 1.28]	3.71 [2.55, 4.87]
Main model + Relative humidity	5.42 [4.81, 6.28]	1.13 [1.08, 1.19]	3.66 [2.81, 4.81]

Notes: Values shown are odds ratio [95% confidence intervals].

*Main model adjusted for all covariates listed in the table, including child characteristics (gender, age, z-score), maternal factors (age, education level), household wealth level, country's gross domestic product per capita, survey year and month, and annual mean temperature.

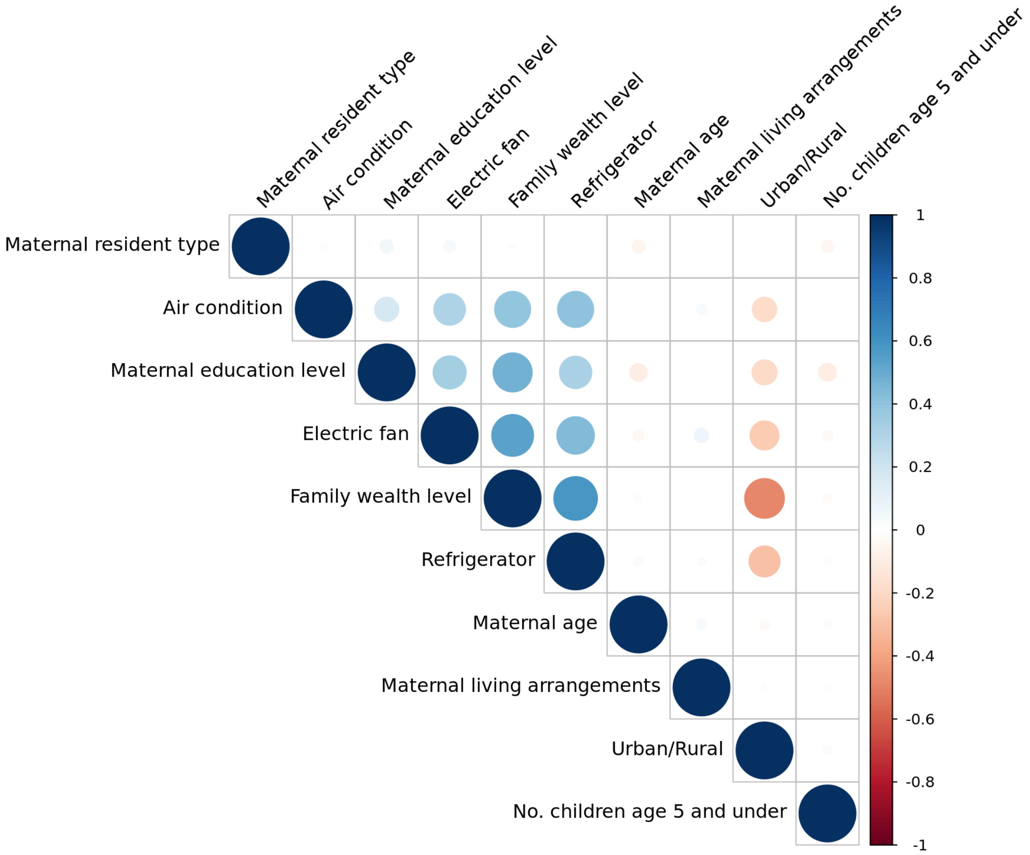

Fig. S1 Spearman correlation matrix of sensitivity and adaptive capacity factors. The size and color intensity of circles represent the strength of correlations. Blue indicates positive correlations and red indicates negative correlations. All correlations were within the range of -0.6 to 0.6, supporting the independence of these factors in subsequent analyses.

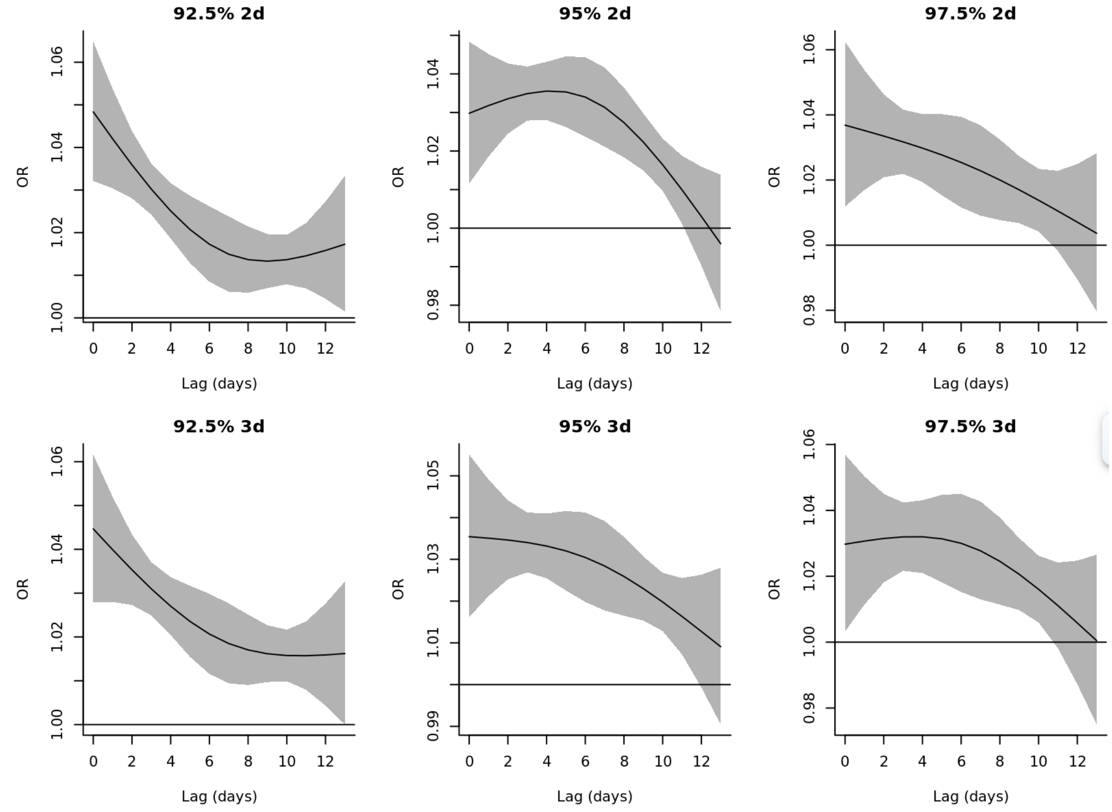

Fig. S2 Lag patterns of heat wave effects on the risk of not meeting Minimum Dietary Diversity (MDD) across different heat wave definitions. Solid lines and shaded areas represent odds ratio and 95% confidence intervals, respectively. Each panel shows the lag-response relationship under different combinations of temperature thresholds (92.5th, 95th, and 97.5th percentiles) and duration criteria (2 days and 3 days).

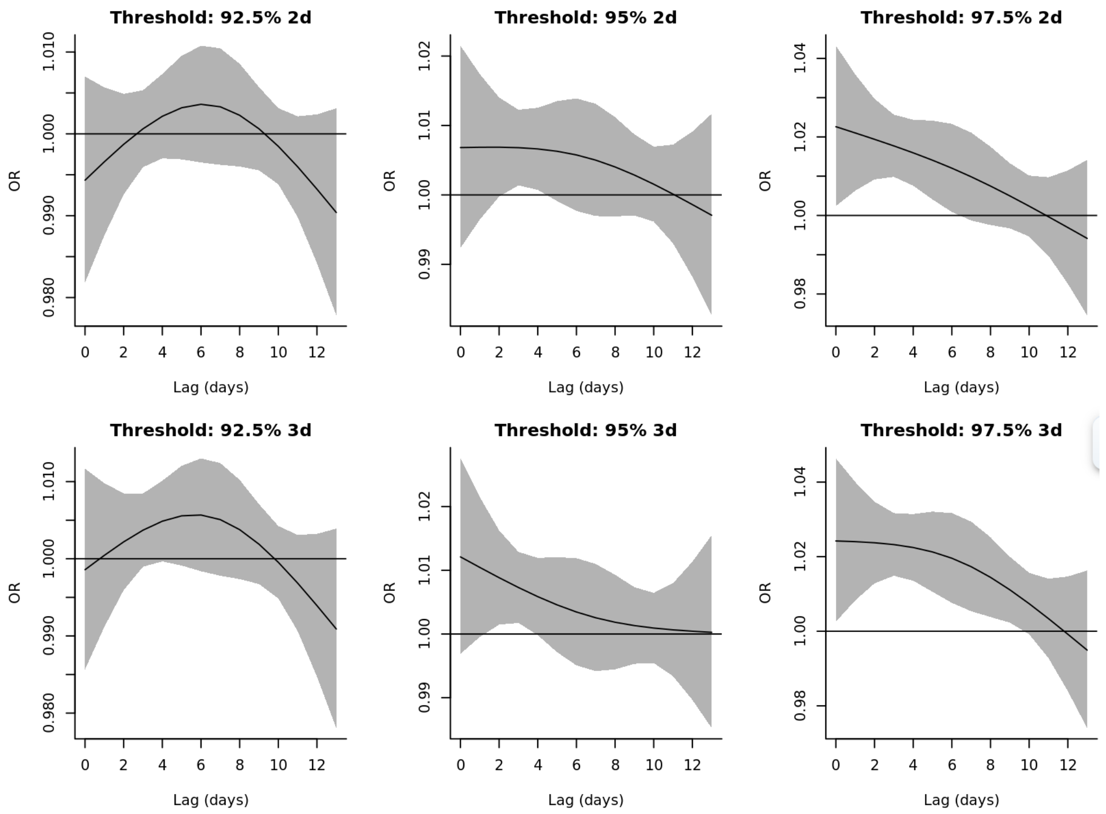

Fig. S3 Lag patterns of heat wave effects on the risk of not meeting Minimum Meal Frequency (MMF) across different heat wave definitions. Solid lines and shaded areas represent odds ratio and 95% confidence intervals, respectively. Each panel shows the lag-response relationship under different combinations of temperature thresholds (92.5th, 95th, and 97.5th percentiles) and duration criteria (2 days and 3 days).

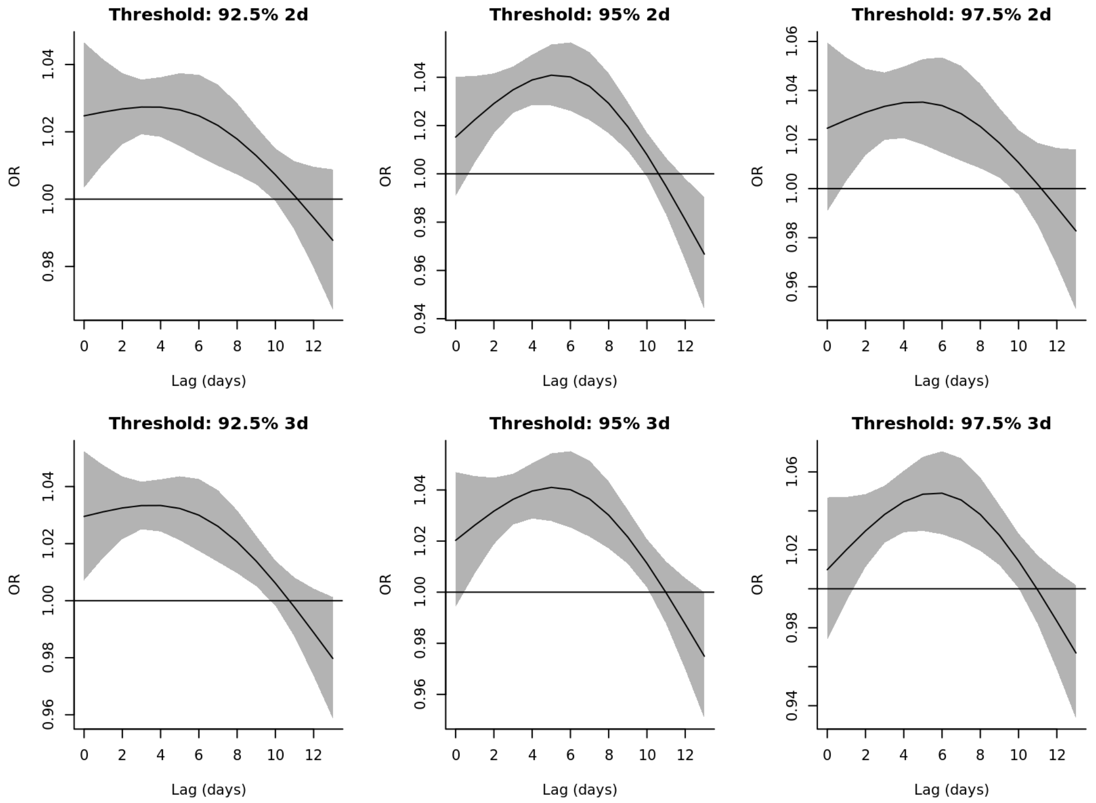

Fig. S4 Lag patterns of heat wave effects on the risk of not meeting Minimum Acceptable Diet (MAD) across different heat wave definitions. Solid lines and shaded areas represent odds ratio and 95% confidence intervals, respectively. Each panel shows the lag-response relationship under different combinations of temperature thresholds (92.5th, 95th, and 97.5th percentiles) and duration criteria (2 days and 3 days).

References

1 Corsi, D. J., Neuman, M., Finlay, J. E. & Subramanian, S. Demographic and health surveys: a profile. *International journal of epidemiology* **41**, 1602-1613 (2012).

2 Rutstein, S. O. & Rojas, G. Guide to DHS statistics. *Calverton, MD: ORC Macro* **38**, 78 (2006).

3 DHS, A. A. O. & DATA, M. M. Dhs methodological reports 13. (2014).

4 Martinez-Millana, A. *et al.* Optimisation of children z-score calculation based on new statistical techniques. *PloS one* **13**, e0208362 (2018).

5 Hersbach, H. *et al.* The ERA5 global reanalysis. *Quarterly Journal of the Royal Meteorological Society* **146**, 1999-2049 (2020).

6 Lawrence, M. G. The relationship between relative humidity and the dewpoint temperature in moist air: A simple conversion and applications. *Bulletin of the American Meteorological Society* **86**, 225-234 (2005).

7 Magnus, G. Versuche über die Spannkräfte des Wasserdampfs. *Annalen der Physik* **137**, 225-247 (1844).
